# The Rise of China in the International Trade Network: A Community Core Detection Approach

**DOI:** 10.1371/journal.pone.0105496

**Published:** 2014-08-19

**Authors:** Zhen Zhu, Federica Cerina, Alessandro Chessa, Guido Caldarelli, Massimo Riccaboni

**Affiliations:** 1 IMT Institute for Advanced Studies Lucca, Lucca, Italy; 2 Department of Physics, Università degli Studi di Cagliari, Cagliari, Italy; 3 Linkalab, Complex Systems Computational Laboratory, Cagliari, Italy; 4 Istituto dei Sistemi Complessi, Consiglio Nazionale delle Ricerche, Rome, Italy; 5 London Institute for Mathematical Sciences, London, United Kingdom; 6 Department of Managerial Economics, Strategy and Innovation, Katholieke Universiteit Leuven, Leuven, Belgium; University of Maribor, Slovenia

## Abstract

Theory of complex networks proved successful in the description of a variety of complex systems ranging from biology to computer science and to economics and finance. Here we use network models to describe the evolution of a particular economic system, namely the International Trade Network (ITN). Previous studies often assume that globalization and regionalization in international trade are contradictory to each other. We re-examine the relationship between globalization and regionalization by viewing the international trade system as an interdependent complex network. We use the modularity optimization method to detect communities and community cores in the ITN during the years 1995–2011. We find rich dynamics over time both inter- and intra-communities. In particular, the Asia-Oceania community disappeared and reemerged over time along with a switch in leadership from Japan to China. We provide a multilevel description of the evolution of the network where the global dynamics (i.e., communities disappear or reemerge) and the regional dynamics (i.e., community core changes between community members) are related. Moreover, simulation results show that the global dynamics can be generated by a simple dynamic-edge-weight mechanism.

## Introduction

*“Befriend a distant state while attacking a neighbor.”*

*Thirty-Six Stratagems*

Theory of complex networks is a modern way to characterize mathematically a series of different systems in the shape of subunits (nodes) connected by their interactions (edges) [1]. Such a modeling proved to be fruitful in the description of a variety of different phenomena ranging from biology [2] to social sciences [3]–[6]. Here we move forward by considering the evolution of the community structure of a particular instance of complex networks. Such an instance is represented by the International Trade Network (ITN), a system composed of the various countries, which are connected by international trade.

The last two decades have witnessed both intensified globalization and regionalization in international trade. The former is evidenced by the formation of unbiased trade relationships across diverse groups of countries while the latter is evidenced by the formation of regional trade agreements and free trade areas. When empirically testing the above two phenomena, previous studies often assume that they are contradictory to each other and try to answer questions like “Has the world become more globalized or regionalized?” Based on various data sets and methodologies, some studies conclude with strong evidence of globalization [7], while others argue the opposite [8], [9], while yet others have mixed results [10].

A fast-growing literature has been built in recent years by viewing the international trade system as an interdependent complex network, where countries are represented by nodes and trade relationships are represented by edges [11]–[17]. As a result, many topics in international economics have been re-investigated through the lens of networks, and globalization and regionalization are certainly no exception. However, even with the networks approach, the question of whether we have a more globalized or regionalized world is still answered with mixed results [18]–[21]. Moreover, the contribution of network analysis to our understanding of international trade has been questioned, since there is still little evidence about the importance of indirect or network effects on the performances of individual countries (nodes) and trade relationships (edges).

In this paper, we re-examine the relationship between globalization and regionalization from a different angle. Instead of assuming that the two are contradictory to each other and attempting to figure out which is dominating the other, we take into account the dynamics in the ITN at both regional level and global level and investigate the interaction between the two. Besides that, we will take advantage of a unique “natural experiment,” that is the opening of China to the world trade and the entry of China in the World Trade Organization in 2001, to analyze the reverberations of a huge country-specific shock on the structure of the ITN.

We make use of the CEPII BACI Database [22] to build up the ITN and use the modularity optimization method [23] to detect both communities and community cores in the ITN during the years 1995–2011. The global dynamics are defined as the disappearance or emergence of the communities over time and the regional dynamics are defined as the leadership (community core) change between community members.

We find that the Asia-Oceania community displayed an interesting interaction between the two, which can be roughly summarized in the following three stages:

1. During 1995–2001, the Asia-Oceania community was present (Only with a brief interruption in 1998, when the Asia-Oceania community was integrated with the America community. Also, during 1999–2001, while China was always a member of the Asia-Oceania community, Japan, Oceania, part of the Southeast Asia, and some other Asian economies were integrated with the America community.) in the ITN and was led by Japan (During 1999–2001, when Japan was integrated with America, the Asia-Oceania community was led by Hong Kong instead.);

2. During 2002–2004, the Asia-Oceania community disappeared and was integrated with the American community, which was led by the United States;

3. During 2005–2011, the Asia-Oceania community reemerged and was led by China.

Our simulation results show that the disappearance and reemergence of the communities can be generated by a dynamic-edge-weight mechanism for both inter- and intra-communities. In a network with a fixed number of nodes and a preset initial community structure, each period a node will be selected and by chance it may increase its edge weight with an inter-community node (if the edge already exists; otherwise a new edge will be established). It will then increase its edge weight with an intra-community neighbor. Those neighbors with more inter-community strength will be preferred. In light of the dynamic-edge-weight mechanism, the rise of China in the Asia-Oceania community can be explained by its dramatic increase of inter-community trade since 2002. The intuition is that, the Asia-Oceania community collapsed after China entered the WTO and built strong trade relationships with other communities, especially with the external cores, i.e., the United States and Germany. China then became regionally attractive and restored the Asia-Oceania community as the community leader after it gained a significant portion of trade globally. Indeed, as quoted in the beginning of the paper, a classical stratagem to achieve regional power is to befriend a distant state. More precisely, the stratagem can be rephrased in the current context as “befriend a distant friend while *attracting* a neighbor.”

Our contribution to the analysis of the ITN is twofold. First, we provide some evidence of a deviation from the Barabási-Albert preferential attachment rule [1], [24] and the law of gravity [25]–[27] in the world trade. Second, we identify a mechanism that can account for this deviation and validate it via simulations and empirical analysis. We show that by increasing its global export China is also increasing the chance to import more goods from regional trading partners. In other words, part of the Chinese export growth shock gets transmitted to other economies in the same region by means of a corresponding increase in Chinese imports of intermediate goods and partial delocalization of production. The transmission mechanism we identify provides further support for a network approach to the analysis of world trade, since we show how local changes in the intensity of trade diffuse to other nodes in the network. We argue that a reductionist approach, which relies exclusively on node and link specific information, misses some important network effects in the world trade structure.

The rest of the paper is structured as follows. Section 2 describes our methodology of community detection and community core detection, respectively. Section 3 summarizes the data we use to build the ITN. The detection results are reported and discussed in Section 4. A model and its simulation results and some empirical evidence to explain the dynamics observed are presented in Section 5. Finally, Section 6 concludes the paper.

## Methodology

### Community Detection

It is well known that one of the main features of networks is community structure, i.e. the partition of a network into clusters, with many (and/or strong) edges connecting nodes in the same cluster and few (and/or weak) edges connecting nodes between different ones [28]. In the following we use the modularity optimization method introduced by Newman and Girvan [23], [29]. It is based on the idea that a random graph is not expected to have a community structure. Therefore, the possible existence of clusters is revealed by the comparison between the actual density of edges in a subgraph and the expected density if the nodes are attached randomly. The expected edge density depends on the chosen null model, i.e., a copy of the original graph keeping some of its structural properties but without community structure [28].

The most popular null model, introduced by Newman and Girvan [23], [29], keeps the degree sequence and consists of a randomized version of the original graph, where edges are rewired at random, under the constraint that the expected degree of each node matches the degree of the node in the original graph.

The modularity function to be optimized is, then, defined as:
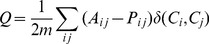
(1)where the summation operator runs over all the node pairs. The 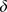 function equals 1 if the two nodes *i* and *j* are in the same community and 0 otherwise. Since the ITN is a weighted network by our formulation, *A* is the weighted adjacency matrix and *A_ij_* is the edge weight between *i* and *j*. 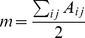 is the total edge weight of the network. Finally, 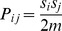 is the probability of the presence of an edge between the two nodes *i* and *j* in the randomized null model and 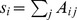 is the strength of node *i*.

*Q* lies in the interval [0,1]. The optimal partition of the network is the community structure with which the maximum value of *Q* is obtained. That is, high value of *Q* indicates more deviations from the random counterpart. The modularity method suffers from various problems, the most important one being the existence of a resolution limit [30], which prevents it from detecting smaller modules. However, it is by far the most used community detection method. It delivers good results and has some nice features such as being a global criterion and simple to implement.

### Community Core Detection

The main drawback of all algorithms for community detection is the fact that they do not provide any information about the importance of any individual node inside the community. Nodes of a community do not have the same importance for the community stability: the removal of a node in the “core” of a network affects the partition much more than the deletion of a node that stays on the periphery of the community [31]. Therefore, in the following we complement community detection with a novel way of detecting cores inside communities by using the properties of the modularity function.

By definition, if the modularity associated with a network has been optimized, every perturbation in the partition leads to a negative variation in the modularity (d*Q*). If we move a node from a partition, we have *M*−1 possible choices (with *M* as the number of communities) as the node's new host community. It is possible to define the 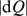 associated with each node as the smallest variation in absolute value (or the closest to 0 since d*Q* is always a negative number) for all the possible choices. This is a measure of how important that node is to its community [31].

It follows that, within a community, the node with higher normalized 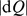 is more important and it is more likely to be the leader of that community. Furthermore, we reason that a more comprehensive leadership indicator of a given node should take into account not only the normalized 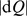 within its community but also the global importance as measured by its strength [32]. Therefore, for node *i*, we calculate the quantity 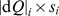 as its leadership indicator, where again 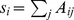 is the strength of node *i*.

Finally, in order to have a better visualization of the relative importance of nodes in different communities we use the so-called *CS* index, ranging from 0 to 1, which is essentially the normalized 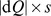 for each community.

## Data

We use the BACI database [22] to build up the ITN. BACI is the world trade database developed by the CEPII at a high level of product disaggregation. Original data are provided by the United Nations Statistical Division (COMTRADE database). BACI is constructed using an original procedure that reconciles the declarations of the exporter and the importer. This harmonization procedure considerably extends the number of countries for which trade data are available, as compared to the original COMTRADE. Furthermore, BACI provides bilateral values and quantities of exports at the HS 6-digit product level, for more than 200 countries since 1995. (See the CEPII website, http://www.cepii.fr/CEPII/en/bdd_modele/presentation.asp?id=1, for further information about BACI.)

We use the BACI database from 1995 to 2011 and, for each year, we sum up all the bilateral commodity flows between any two countries. We construct the ITN with countries as nodes and with the total bilateral trade flow between countries *i* and *j* as the edge weight *A_ij_*.

## Community Detection Results

### Global Dynamics versus Regional Dynamics

During the years 1995–2011 we have examined, the ITN was mainly characterized by three communities, namely, the America community, the Europe community, and the Asia-Oceania community. According to the United Nations definitions of macro geographical regions (See the website of the United Nations Statistics Division, https://unstats.un.org/unsd/methods/m49/m49regin.htm.), the America community is more or less comprised of Americas. The Europe community is more or less comprised of Europe and Central Asia. The Asia-Oceania community is more or less comprised of Eastern Asia, Southern Asia, South-Eastern Asia, and Oceania. (Countries in Africa and Western Asia don't have consistent community memberships over time. Therefore, they are not classified in any of the three communities.)

However, among the three main communities, the America community and the Europe community were more stable than the Asia-Oceania community. First, over the 17 years, the America community and the Europe community were always present while the Asia-Oceania community experienced disappearance and reemergence. Second, the intra-community structure was more stable in the America community and the Europe community in a sense that the community leaders (cores) over time were always the United States and Germany, respectively. The Asia-Oceania community on the other hand experienced a leadership change from Japan to China.

Because the Asia-Oceania community has shown rich dynamics both internally and externally, in Subsection 4.2 we focus our attention on it.

### The Asia-Oceania Community

As mentioned in Section 1, the dynamics of the Asia-Oceania community can be roughly divided into three stages, namely, its presence with Japan's leadership during 1995–2001, its disappearance and integration with the America community during 2002–2004, and finally its reemergence with China's leadership during 2005–2011.

**Figure 1:**
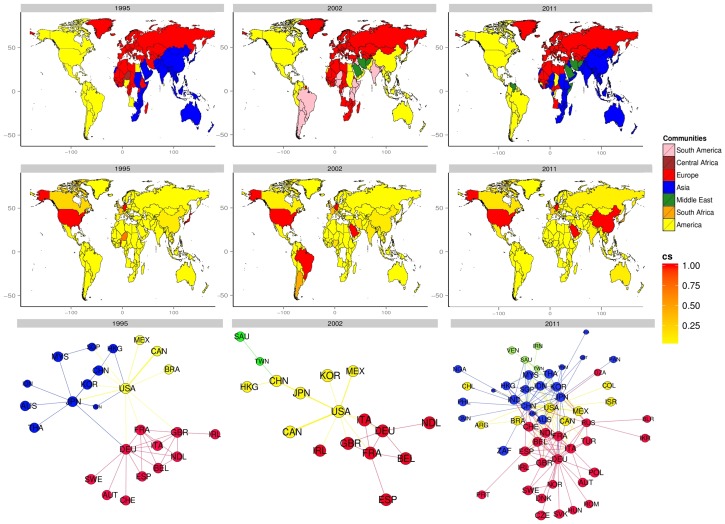
Community and Community Core Detection Results. From left to right, the three columns are corresponding to the years 1995, 2002, and 2011, respectively. The first row shows the Newman-Girvan community detection results. The America community is colored yellow, the Europe community is colored red, and the Asia-Oceania community is colored blue. Asia-Oceania and America were separated from each other in 1995 and 2011 but was integrated in 2002. The second row shows the community core detection results by normalizing 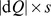 for each community. The redness of each country is proportional to its relative magnitude of 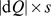 within its community (*CS*). The reddest country in the Asia-Oceania community was Japan back in 1995 but became China in 2011. Finally, the third row provides a topological view of the community structure in the three years. Only the edges with no less than 10 million US dollars are shown. Again, Japan was central in the Asia-Oceania community in 1995 and it was replaced by China in 2011.

The same pattern is shown in Figure 1, where three years, 1995, 2002, and 2011, are selected to represent the three stages respectively. (The results for all years from 1995 to 2011 are provided in the supporting information. See Figure S1 for the community detection results for all years and Figure S2 for the community core detection results for all years.) The first row shows the community maps in the three years. The America community is colored yellow, the Europe community is colored red, and the Asia-Oceania community is colored blue. Notice that in 2002 the blue community was by and large merged with the yellow community. (Another interesting change in the world trade community structure is the emergence of the Arab community after 2001. This interesting phenomenon deserves further scrutiny in future research.) The second row shows the community core detection results for the three years. The redder the more important the country is in reserving its community. Equivalently, the yellower the less important the country is in reserving its community. This can be used to identify the leaders in the communities. Notice that in 1995 the reddest country in the Asia-Oceania community was Japan while in 2011 China became the reddest. Finally, the third row provides a topological view of the community structure in the three years. Again, Japan was central in the Asia-Oceania community in 1995 and it was replaced by China in 2011.

## The Linkage Between Global and Regional Network Dynamics

Given its breathtaking economic growth during 1995–2011, it is not surprising to see China's rise in the regional trade community. The rationale behind is the long-established gravity model of trade [25]–[27]. That is, the increased economic mass of China tends to attract more trade flows with other economies. What remains unexplained, however, is the fact that the leadership change from Japan to China is correlated with the disappearance and reemergence of the Asia-Oceania community.

The dynamics observed in the Asia-Oceania community also differ from the prediction of the Barabási-Albert preferential attachment model [1], [24]. According to the preferential attachment mechanism, when choosing another community member with whom the edge weight is to be increased, the given node will prefer the candidates with higher strength. If this is the case, the leadership of Japan in the Asia-Oceania community should be reinforced given that its strength was well ahead of China before 2000. However, Japan was later replaced by China as the community leader. Therefore, we conjecture that not only the magnitude of strength matters but the attributes of nodes such as size and distance also matter in the process of network growth. Moreover, instead of mechanically following an attachment rule, any economic agent plays strategically in choosing its partner to interact with [33], [34]. Finally, unlike the assumption of the preferential attachment model, countries often have limited resources and competences and cannot freely choose trading partners.

To account for the linkage between the global dynamics and the regional dynamics, we propose a simple dynamic-edge-weight mechanism for both inter- and intra-communities.

### A Simple Mechanism for Both Inter- and Intra-Communities

Since the number of countries in the ITN is constant over time and the evolution of the ITN is only concerned with the trade flows between countries, our model is therefore based on a fixed number of nodes and a dynamic-edge-weight mechanism for both inter- and intra-communities. (There exists some related literature to our model. For example, Barrat et al. [35] and Riccaboni and Schiavo [14] examine the network evolution with dynamic edge weights. Li and Maini [36] investigate the network properties with a preferential attachment mechanism for both inter- and intra-communities. However, to the best of our knowledge, our model is the first attempt to bring the dynamics both inter- and intra-communities to the context of a weighted network with a fixed number of nodes.) Additionally, our model is based on an undirected network because the ITN is constructed by total bilateral trade flows.

The initial status of the network is characterized by *M* arbitrarily imposed local communities. (In the context of ITN, the communities can be formed, for instance, by continents.) For simplicity, each community has the same number of nodes, *m*_0_. As a subgraph, each community is completely connected with a equal edge weight, i.e., every node is connected with every node by the same edge weight in the community. Between any two communities, there is only one edge connecting two randomly selected nodes in the two communities respectively. Again for simplicity, the inter-community edge weight is set to equal the initial intra-community edge weight. After the initial set-up, each period the dynamic-edge-weight mechanism is comprised of the following steps:

1. One node, *i*, is randomly selected based on a uniform distribution across all the nodes in the network;

2. Suppose that *i* belongs to community *j*, by chance, *i* can increase its edge weight with a node outside community *j*. And the reach-out probability is 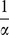, where 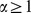 and a big 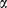 (In the context of the ITN, a high value of 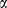 can be interpreted as trade barriers such as tariffs, transportation costs, and language difference.) means that any node will have low probability to reach out to other communities;

3. There are (*M*−1)*m*_0_ nodes outside community *j*. They are equally likely to be chosen by *i* to increase the mutual edge weight. After the inter-community node is identified, the mutual edge weight will be increased by 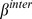;

4. The next step for *i* is to choose a neighbor in the same community *j* to increase the edge weight. The neighbor is selected by the following probability mass function:
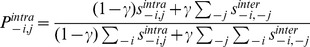
(2)where −*i* is a neighbor to *i* in the community *j* and −*j* is a community other than community *j*. 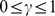 and when 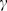 gets close to 1, although *i* prefers to increase the edge weight with the neighbors with more intra-community strength, it prefers even more the ones with more inter-community strength. After the neighbor is identified, the mutual edge weight will be increased by 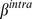;

5. Finally, the modularity optimization method is used to detect the community structure, which may deviate from the original set-up.

### Simulation Results

The initial status of our simulation is a network with 3 preset communities. Each community has 5 nodes and, as mentioned above, each community is completely connected and there is a single edge between any two communities. Other model parameters are 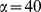, 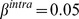, and 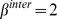, respectively. Setting *alpha* to 40 and having a relatively big 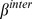 compared to 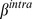 are to make it difficult for a node to reach out to other communities so that the preset community structure can be restored over time. However, when a node does reach out, it is enough to introduce a perturbation to the community structure. Finally, we vary the value of 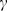 from 0.1 to 0.9 with the step size of 0.05.

**Figure 2:**
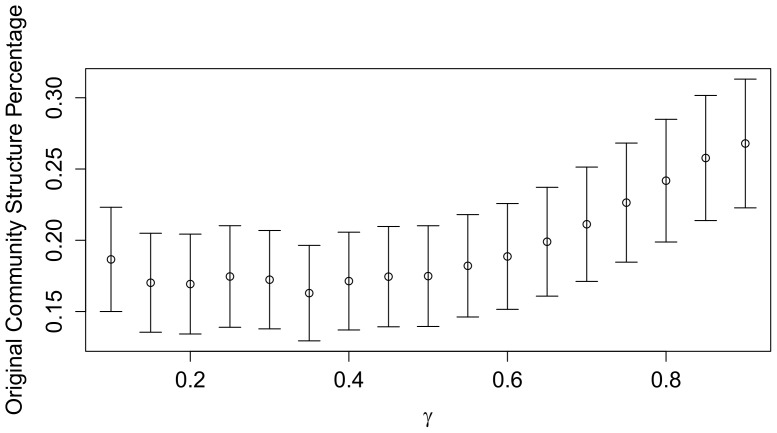
Simulation Result. The 95% confidence interval is calculated for each value of 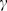 from 0.1 to 0.9 with the step size of 0.05 (the x-axis). The simulation is based on a dynamic-edge-weight mechanism for both inter- and intra-communities. Other model parameters are 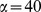, 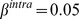, and 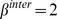, respectively. We define a trial of simulation as running the dynamic-edge-weight mechanism for 5000 periods. As an indicator of the community structure stability of the network, the y-axis is the percentage of the number of the periods with exactly the same community structure as the initial status out of the 5000 periods. Finally, for each value of 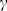, we estimate the confidence interval of the original community structure percentage by collecting a 100-trial sample.

We define a trial of simulation as running the above dynamic-edge-weight mechanism for 5000 periods. We also calculate the percentage of the number of the periods with exactly the same community structure as the initial status out of the 5000 periods as an indicator of the community structure stability of the network. For each value of 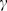, we collect a sample size of 100 trials to compute the 95% confidence interval of the estimated original community structure percentage. The result is reported in Figure 2. As expected, putting more weight on the neighbors with more inter-community strength (i.e., bigger 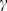) tends to make the community structure more stable (i.e., bigger original community percentage). The intuition is that the reaching-out nodes will be dragged back to their original communities by the preference for their growing inter-community strength.

**Figure 3:**
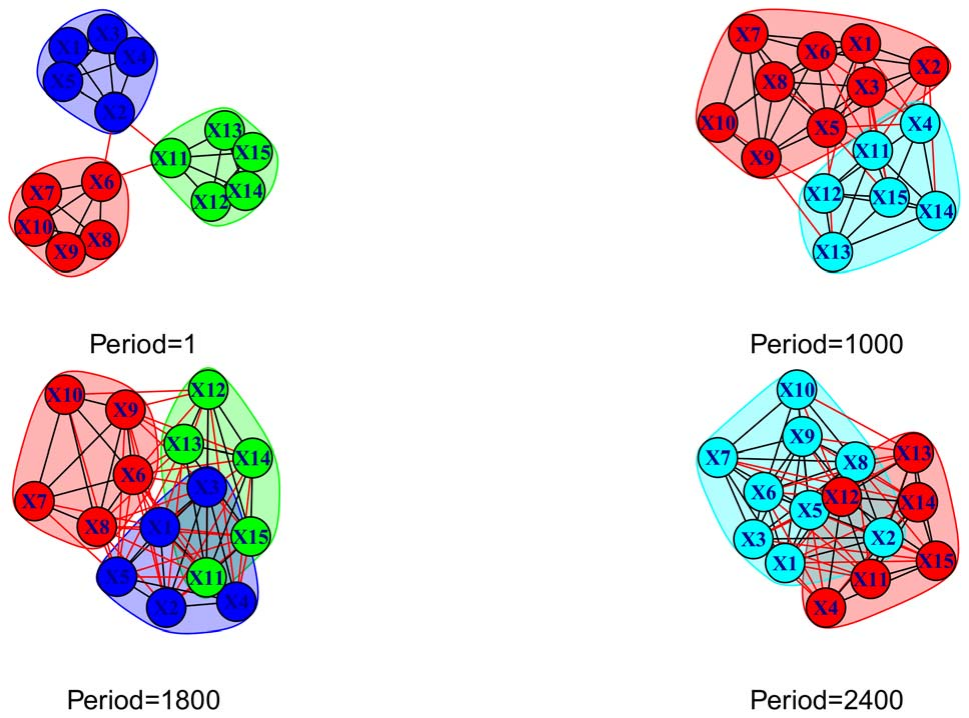
A Detailed Example. The figure is based on a single trial of simulation. Different colors represent different communities detected by the Newman-Girvan method. The inter-community edges are colored red while the intra-community ones are colored black. Although the community detection takes into account the edge weights, all the edges in the figure have the same width. In period 1, three predetermined communities, X1-X5, X6-X10, and X11-X15, are imposed in the network. The number of communities detected in this 15-node network bounces back and forth between 3 and 2 during the simulated periods. That is, like what we observe from the ITN, the disappearance and reemergence of the communities can be generated by the dynamic-edge-weight mechanism for both inter- and intra-communities.

As a detailed example of the simulation, Figure 3 selects 4 periods of a single trial. The 3 preset communities are X1-X5, X6-X10, and X11-X15, respectively. Different colors represent different communities detected by the modularity optimization method. The red edges are inter-community ones while the black ones are intra-community. Like what we observe from the ITN, the disappearance and reemergence of the communities can be generated by the dynamic-edge-weight mechanism for both inter- and intra-communities.

### Empirical Evidence

We now turn back to the ITN and present some empirical evidence for the dynamic-edge-weight mechanism for both inter- and intra-communities.

**Figure 4:**
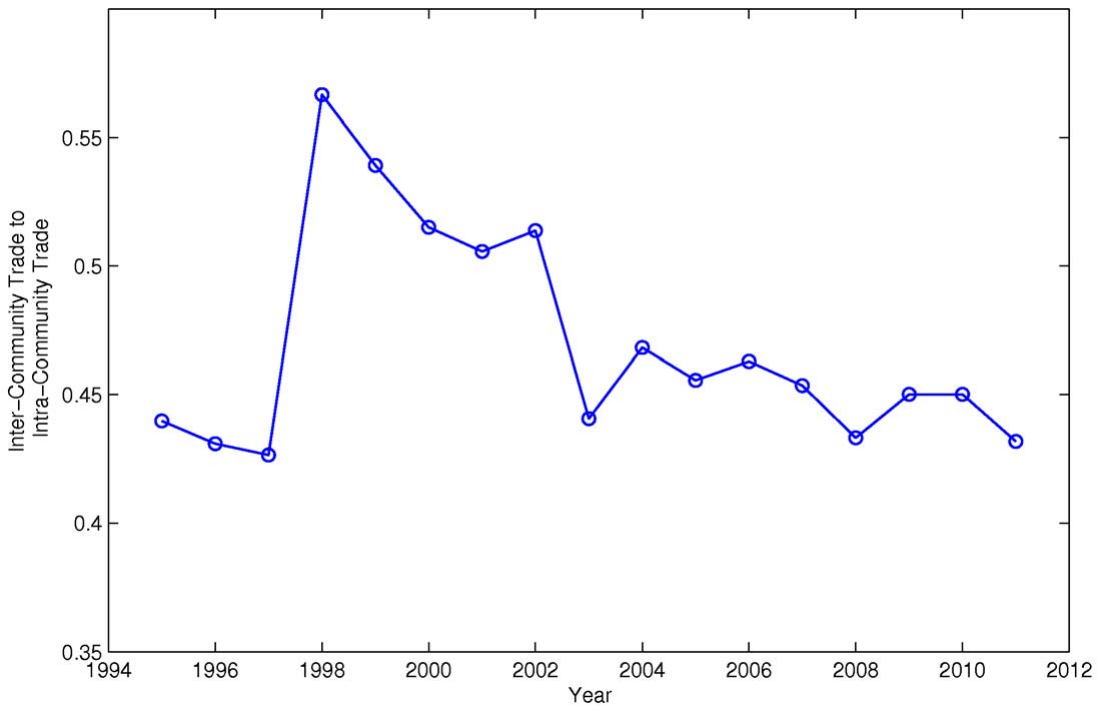
Inter- versus Intra-Community Trade Ratio between Asia-Oceania and America. We calculate the ratio of the inter-community trade to the intra-community trade between the Asia-Oceania community and the America community. The ratio first went up and then went down and formed a hump shape over time. This finding coincides with the disappearance and reemergence of the Asia-Oceania community observed in Figure 1.

First, for the inter-community dynamics, we calculate the ratio of the inter-community trade to the intra-community trade between the Asia-Oceania community and the America community. As shown in Figure 4, the ratio first went up and then went down and formed a hump shape over time. This finding coincides with the disappearance and reemergence of the Asia-Oceania community observed in Figure 1. In 1995, when the Asia-Oceania community was present, the inter-community trade between Asia-Oceania and America was about 44% of the intra-community trade within the two communities. In 2002, when the Asia-Oceania community disappeared, the ratio went up to about 51%. Finally, the ratio went back to about 43% in 2011, when the Asia-Oceania community was present again.

**Figure 5:**
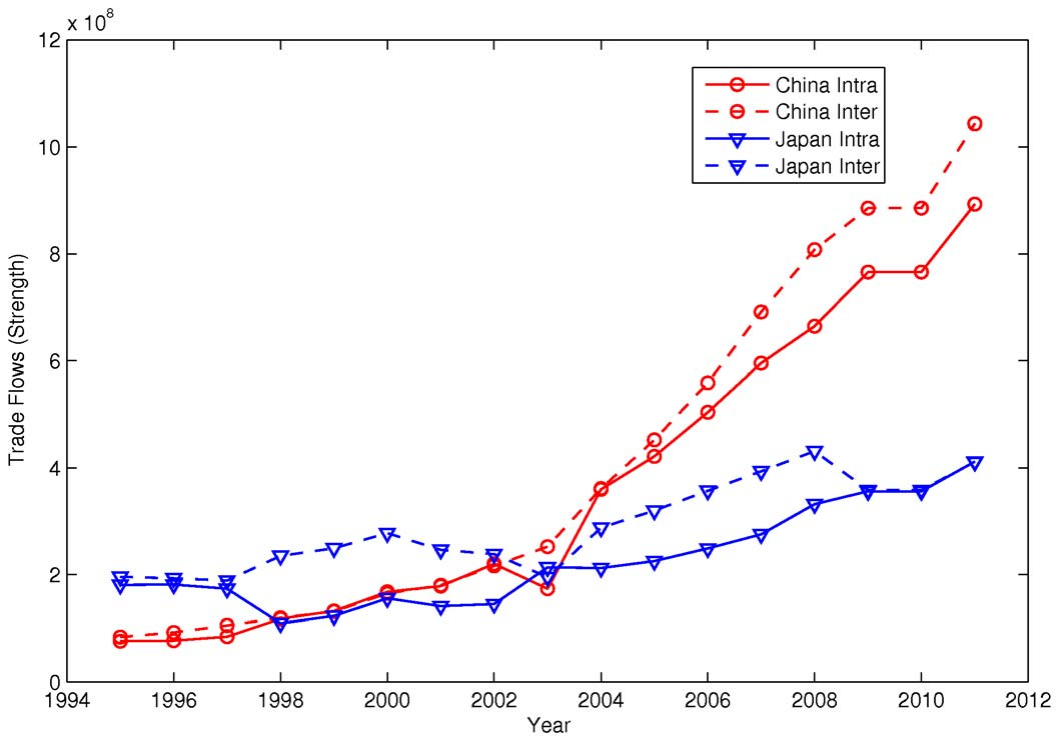
Intra- and Inter-Community Strength of Japan and China. We calculate both the inter- and intra-community trade volumes for Japan and China. Japan had more inter-community trade than China before 2003. However, after 2003, China surpassed Japan in terms of both inter- and intra-community trade. This finding coincides with the leadership change from Japan to China observed in Figure 1. Furthermore, for both countries, the intra-community trade follows closely to the inter-community trade, which can be viewed as evidence of the intra-community dynamic-edge-weight mechanism.

Second, for the intra-community dynamics, we compare the intra-community strength and the inter-community strength between Japan and China. As shown in Figure 5, before 2003, Japan always had more inter-community trade than China and had more intra-community trade in the beginning and slightly less later. After 2003, China surpassed Japan in terms of both inter- and intra-community trade. This finding coincides with the leadership change from Japan to China observed in Figure 1. Also notice that, for both countries, the intra-community trade follows closely to the inter-community trade, which can be considered as evidence of the intra-community dynamic-edge-weight mechanism.

We also check the regional trade agreements (RTAs) for the intra-community dynamics. Table 1 summarizes the effective RTAs signed with China during 1995–2011. Only after its accession to WTO in the end of 2001, China started to form RTAs in 2003 and with countries almost exclusively in the Asia-Oceania community.

**Table 1:** China's Effective RTAs.

RTA Name	Date of Entry into Force
China - Hong Kong, China	29-Jun-2003
China - Macao, China	17-Oct-2003
ASEAN - China	01-Jan-2005(G); 01-Jul-2007(S)
Chile - China	01-Oct-2006(G); 01-Aug-2010(S)
Pakistan - China	01-Jul-2007(G); 10-Oct-2009(S)
China - New Zealand	01-Oct-2008
China - Singapore	01-Jan-2009
Peru - China	01-Mar-2010
China - Costa Rica	01-Aug-2011

This table has all the effective RTAs involving China during 1995–2011. (G) stands for Goods and (S) for Services. The data is extracted from the WTO website, http://rtais.wto.org/UI/PublicAllRTAList.aspx.

Last but not least, it is a well observed fact that the Asia-Oceania community is an active participant of the global production chain (or global value chain) [37]–[39]. Therefore, the intra-community preference over the nodes with more inter-community strength can be understood as the incentive to have better market access through the regional big player in the global production chains.

## Concluding Remarks

By viewing the international trade system as an interdependent complex network and China's opening to world trade as a natural experiment, this paper uses community detection and community core detection techniques to examine both the global dynamics, i.e., communities disappear or reemerge, and the regional dynamics, i.e., community core changes between community members, in the ITN over the period from 1995 to 2011. We find that the Asia-Oceania community has displayed rich dynamics both internally and externally. That is, the Asia-Oceania community was present during 1995–2001 and was led by Japan, and then it disappeared and was integrated with the America community during 2002–2004, and finally it reemerged during 2005–2011 and was led by China.

With a model of a dynamic-edge-weight mechanism for both inter- and intra-communities, we are able to explain the dynamics observed in the Asia-Oceania community. In a network with a fixed number of nodes and a preset initial community structure, each period a node will be selected and by chance it may increase its edge weight with an inter-community node (if the edge already exists; otherwise a new edge will be established). It will then increase its edge weight with an intra-community neighbor. Those neighbors with more inter-community strength will be preferred. Our simulation results show that the global dynamics, i.e., communities disappear or reemerge can be generated by this model setting.

In light of this simple mechanism, the interpretation of the dynamics in the Asia-Oceania community can be that, the community collapsed after China entered the WTO and built strong trade relationships with other communities, especially with the external cores, i.e., the United States and Germany, and China became regionally attractive due to the preference of external strength and restored the Asia-Oceania community as the community leader.

We find some supporting evidence in the trade data. In particular, the behavior of the ratio of the inter-community trade to the intra-community trade between the Asia-Oceania community and the America community coincides with the disappearance and reemergence of the Asia-Oceania community. Within the community, China surpassed Japan after 2003 in terms of both inter- and intra-community trade. In our simulation, the external strength can only be increased by chance. In reality, however, it can be achieved by a series of strategic moves in trade policy. This is evidenced by the surging number of RTAs that China formed since 2003. Moreover, the intra-community preference of the nodes with more inter-community strength can be understood as the incentive to have better market access through the regional big player in the global production chains.

## Supporting Information

Figure S1 **Community Detection Results for All Years.** Here we show the Newman-Girvan community detection results for the ITN during 1995–2011. The America community is colored yellow, the Europe community is colored red, and the Asia-Oceania community is colored blue. During 1995–2001, the Asia-Oceania community was present (only with a brief interruption in 1998, when the Asia-Oceania community was integrated with the America community). During 2002–2004, the Asia-Oceania community disappeared and was integrated with the American community. Finally, during 2005–2011, the Asia-Oceania community reemerged.
(PDF)

Figure S2
**Community Core Detection Results for All Years.** Here we show the community core detection results during 1995–2011 by normalizing 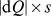 for each community. The redness of each country is proportional to its relative magnitude of 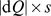 within its community (*CS*). During 1995–2001, the Asia-Oceania was mostly led by Japan (except for 1999–2001, when Japan was integrated with America, the Asia-Oceania community was led by Hong Kong instead). During 2002–2004, the Asia-Oceania community disappeared and was integrated with the American community, which was led by the United States. During 2005–2011, the Asia-Oceania community reemerged and was led by China.(PDF)
