# Supplementary figures and images for: The Rise of China in the International Trade Network: A Community Core Detection Approach
Source: PLoS One. 2014 Aug 19;9(8):e105496. doi: 10.1371/journal.pone.0105496 (PMC4138169; doi:10.1371/journal.pone.0105496)

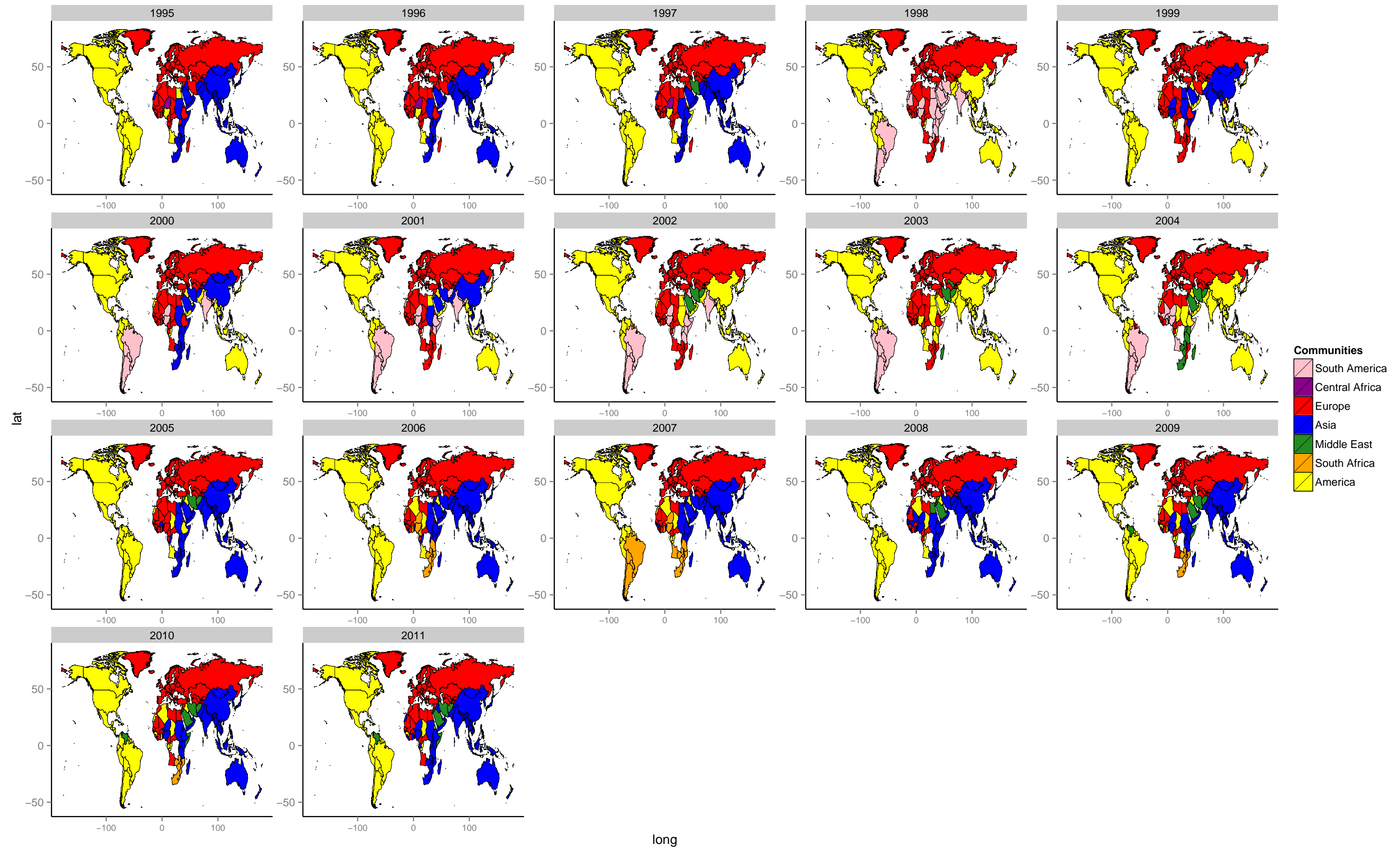

Supplement: Figure S1 — Community Detection Results for All Years. Here we show the Newman-Girvan community detection results for the ITN during 1995–2011. The America community is colored yellow, the Europe community is colored red, and the Asia-Oceania community is colored blue. During 1995–2001, the Asia-Oceania community was present (only with a brief interruption in 1998, when the Asia-Oceania community was integrated with the America community). During 2002–2004, the Asia-Oceania community disappeared and was integrated with the American community. Finally, during 2005–2011, the Asia-Oceania community reemerged. (PDF) [file pone.0105496.s001.pdf]

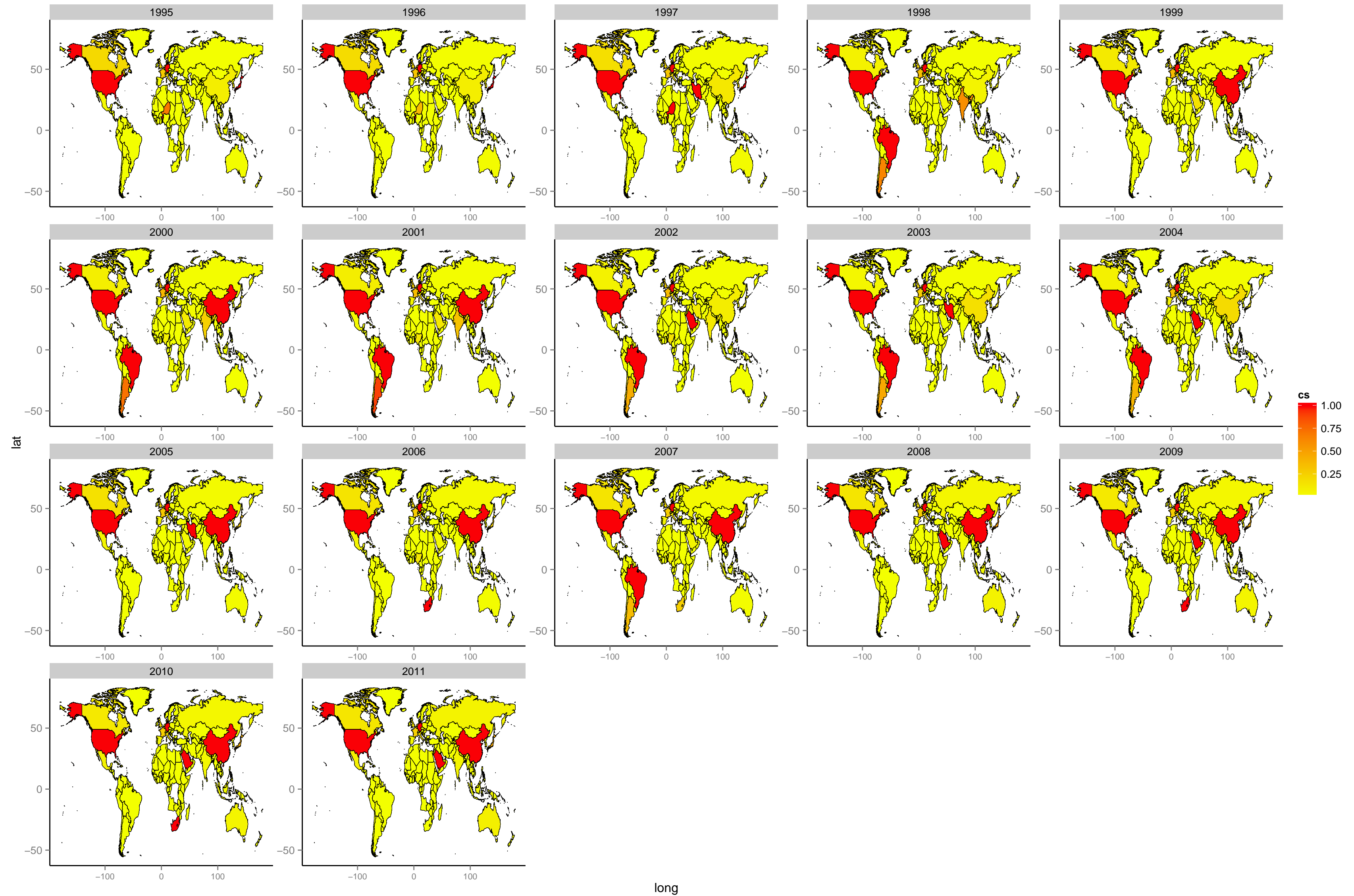

Supplement: Figure S2 — Community Core Detection Results for All Years. Here we show the community core detection results during 1995–2011 by normalizing for each community. The redness of each country is proportional to its relative magnitude of within its community (CS). During 1995–2001, the Asia-Oceania was mostly led by Japan (except for 1999–2001, when Japan was integrated with America, the Asia-Oceania community was led by Hong Kong instead). During 2002–2004, the Asia-Oceania community disappeared and was integrated with the American community, which was led by the United States. During 2005–2011, the Asia-Oceania community reemerged and was led by China. (PDF) [file pone.0105496.s002.pdf]
